# Molecular Dynamics of Sialic Acid Analogues and their Interaction with Influenza Hemagglutinin

**DOI:** 10.4103/0250-474X.73919

**Published:** 2010

**Authors:** T. Femlin Blessia, V. S. Rapheal, D. J. S. Sharmila

**Affiliations:** Department of Bioinformatics, Karunya University, Karunya Nagar, Coimbatore-641 114, India; 1 Department of Biotechnology, Kumaraguru College of Technology, Coimbatore-641 006, India

**Keywords:** AMBER, molecular dynamics, molecular mechanics, molecular modeling, sialic acid analogues

## Abstract

Synthetic sialic acid analogues with multiple modifications at different positions(C-1/C-2/C-4/C-8/C-9) are investigated by molecular mechanics and molecular dynamics to determine their conformational preferences and structural stability to interact with their natural receptors. Sialic acids with multiple modifications are soaked in a periodic box of water as solvent. Molecular mechanics and a 2 nanosecond molecular dynamics are done using amber force fields with 30 picosecond equilibrium. Direct and water mediated hydrogen bonds existing in the sialic acid analogues, aiding for their structural stabilization are identified in this study. The accessible conformations of side chain linkages of sialic acid analogues holding multiple substituents are determined from molecular dynamics trajectory at every 1ps interval. Transitions between different minimum energy regions in conformational maps are also noticed in C-1, C-2, C-4, C-8 and C-9 substituents. Docking studies were done to find the binding mode of the sialic acid analogues with Influenza hemagglutinin. This finding provides stereo chemical explanation and conformational preference of sialic acid analogues which may be crucial for the design of sialic acid analogues as inhibitors for different sialic acid specific pathogenic proteins such as influenza toxins and neuraminidases.

The binding of a virus particle to the surface receptors of a host cell is mediated by viral proteins, which specifically recognize receptor determinants, such as peptides, lipids or carbohydrates[1]. The receptor determinants of Influenza virus are shown to be sialic acid residues, which constitute more than 20 naturally occurring derivatives of neuraminic acid collectively referred to as a family of sialic acids. These are found widely distributed in animal tissues and in bacteria, especially in glycoproteins and gangliosides. Influenza A and B bind to the most abundant sialic acid, N-acetyl neuraminic acid (Neu5Ac or NANA). The binding of influenza A virus is mediated by the major virus surface glycoprotein, hemagglutinin (HA) that binds to the terminal sialic acid residues as the first step of viral infection and mediates both the initial attachment of virus to target cells and the subsequent fusion of the viral and cell membranes[2]. The region of the HA involved in receptor binding has been deduced from studies of mutant HAs with different binding specificities to involve a pocket of amino acids at its membrane distal surface[3]. The three-dimensional structure of influenza virus HAs complexed with cell receptor analogues show neuraminic acid bound to this pocket fundamentally through hydrogen bonds and van der Waals interactions[4].

Based on this knowledge, it should, in principle be possible to find a neuraminic acid analogue that mimics the cell receptor and thus preferentially binds to the virus, thereby blocking attachment. One approach in the design of high-affinity inhibitors is to use Neu5Ac (or its 2α-O-methyl derivative) as a scaffold and to modify its functional groups in order to increase its affinity for the HA. The configuration of neuraminic acid places the carboxylate in the axial position is the alpha-anomer of neuraminic acid.

Present work was initiated with the modeling of the alpha-anomer of neuraminic acid and its derivatives having multiple substitutions at C-1, C-2, C-4, C-8 and C-9 positions. Molecular mechanics and molecular dynamics calculations were performed. The conformational behaviour of varying substituent holding side chains of neuraminic acid in aqueous environment were studied. The direct and water-mediated hydrogen bonds, which played a major role in the structural stability of neuraminic acid were also analyzed. Docking studies were done to study the binding mode of neuraminic acid derivatives into the binding pocket of Influenza HA.

## MATERIALS AND METHODS

The modeled neuraminic acid derivatives with multiple substituents are shown in Table 1. The neuraminic acid derivatives are designed as described by Sauter, *et al*., 1992[5] and Bianco, *et al*., 1998[6]. The initial conformations of the different substituent holding side chains of neuraminic acid analogues are defined as follows: for C-1 substitution, γ_1_=0 when C3-C2 cis to C1-O1A; γ_2_=0 when C2-C1 cis to O1A-C11, where C11 is the carbonyl carbon atom from the substitution group. For C-2 substitution, θ_1_=0 when C4-C3 cis to C2-O2; θ_2_=0 when C3-C2 cis to O2-C12, where C12 is the carbon attached to the substituent group. For C-4 substitution, β_1_=0 when C2-C3 cis to C4-O4; β_2_=0 when C3-C4 cis to O4-C13, where C13 is the carbon atom attached to the substituent group. For C-8 substitution, χ_1_=0 when C5-C6 cis to C7-C8; χ_3_=0 when C6-C7 cis to C8-O8. For C-9 substitution, χ_1_=0 when C5-C6 cis to C7-C8; χ_2_=0 when C6-C7 cis to C8-C9.

**TABLE 1:** NEURAMINIC ACID ANALOGUES WITH MULTIPLE MODIFICATIONS

Sialic acid derivative	Substituents				
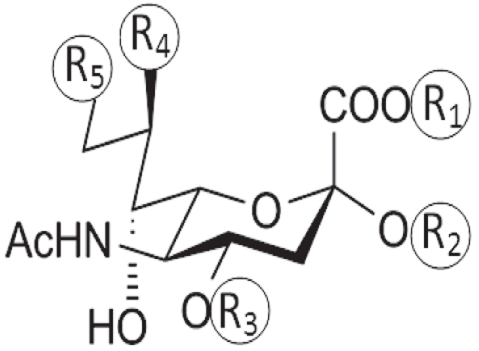	R_1_	R_2_	R_3_	R_4_	R_5_

N-acetyl Neuraminic Acid	H	H	H	OH	OH
methyl 5-N-acetyl neuraminate	CH_3_	H	H	OH	OH
methyl 2α-O-methyl-5-N-acetyl neuraminate	CH_3_	CH_3_	H	OH	OH
benzyl 2α-O-methyl-5-N-acetyl-8,9-O-isopropylidene neuraminate	CH_2_Ph	CH_3_	H	OCH_3_	OCH_3_
benzyl 2α-O-methyl-4-O-capriloyl-5-N-acetyl-8,9-O-isopropylidene neuraminate	CH_2_Ph	CH_3_	CO_2_(CH_2_)_6_CH_3_	OCH_3_	OCH_3_
benzyl 2α-O-methyl-4-O-capryloil-5-N-acetyl neuraminate	CH_2_Ph	CH_3_	CO_2_(CH_2_)_6_CH_3_	OH	OH
2α-O-methyl-4-O-capriloyl-5-N-acetyl neuraminic acid	H	CH_3_	CO_2_(CH_2_)_6_CH_3_	OH	OH
benzyl-2α-O-methyl-4-O-(8-morpholin)-capriloyl-5-N-acetyl-8,9-Oisopropylidene-neuraminate	CH_2_Ph	CH_3_	CO_2_(CH_2_)_7_O(CH_2_CH_2_)_2_NH	OCH_3_	OCH_3_
benzyl-2α-O-methyl-4-O-(8-morpholin)-capriyloyl-5-N-acetylneuraminate	CH_2_Ph	CH_3_	CO_2_(CH_2_)_7_O(CH_2_CH_2_)_2_NH	OH	OH
2α-O-methyl-4-O-(8-morpholin)-capriloyl-5-N-acetyl neuraminic acid	H	CH_3_	CO_2_(CH_2_)_7_O(CH_2_CH_2_)_2_NH	OH	OH
5-N-acetyl-9-amino-9-deoxy neuraminic acid	H	H	H	OH	NH_2_

Molecular mechanics calculations were carried out in the Pentium IV workstation using SANDER module of software AMBER10[7]. The force fields AMBER ff03 and gaff (general amber force field) were used. Water molecules are added from the solvent library of AMBER10 and care is given to maintain the number of water molecules to be same for all the neuraminic acid derivatives.

A periodic box enclosing the neuraminic acid analogues in solution is constructed to turn it into a periodic system for the simulation programs and periodic boundary conditions are applied on constant volume (Table 2). The non bonded pair list was updated for every 10 steps. The non-bonded cutoff was specified as 8Å. For initial 100 cycles steepest descent method was used, then conjugate gradient is switched on. The convergence criterion for the energy gradient is less than 0.01kcal mole^-1^. The energy minimized structures for all the neuraminic acid derivatives are intensively analyzed through graphics software VEGA ZZ to work out the direct and solvent mediated hydrogen bonds.

**TABLE 2:** THE BOX SIZE AND NUMBER OF ATOMS IN EACH NEURAMINIC ACID ANALOGUE WITH MULTIPLE MODIFICATIONS

Sialic acid derivative	No of atoms	Box size(Å^3^)
N-acetyl Neuraminic Acid	40	25.071×22.641×23.266
methyl 5-N-acetyl neuraminate	43	23.338×22.854×24.077
methyl 2α-O-methyl-5-Nacetyl neuraminate	46	24.734×23.343×22.600
benzyl 2α-O-methyl-5-N-acetyl-8,9-Oisopropylidene neuraminate	62	24.734×23.343×24.077
benzyl 2α-O-methyl-4-O-capriloyl-5-N-acetyl-8,9-O-isopropylidene neuraminate	85	21.402×30.414×21.022
benzyl 2α-O-methyl-4-O-capryloil-5-N-acetyl neuraminate	79	21.402×29.461×21.362
2α-O-methyl-4-Ocapriloyl-5-N-acetyl neuraminic acid	66	23.338×21.568×26.582
benzyl-2α-O-methyl-4-O-(8-morpholin)-capriloyl-5-N-acetyl-8,9-O-isopropylideneneuraminate	98	20.772×32.158×21.362
benzyl-2α-O-methyl-4-O-(8-morpholin)-capriyloyl-5-N-acetyl-neuraminate	92	20.086×30.511×23.266
2α-O-methyl-4-O-(8-morpholin)-capriloyl-5-Nacetyl neuraminic acid	79	25.071×20.106×29.273
5-N-acetyl-9-amino-9-deoxy neuraminic acid	41	25.002×20.983×24.417

To understand the conformational dynamics of the neuraminic acid analogues in aqueous environment, molecular dynamics calculations were performed over a period of 30ps equilibration followed by a 2 ns production run with explicit inclusion of water molecules. The width of integration step of the MD simulation was 1fs. The history of information was recorded for every 1000 steps of trajectory which resulted in 2000 structures. The temperature was maintained to be 300K. The total simulation time was around 17 h for each molecule. The MD trajectory information collected for every 1ps were analyzed using PTRAJ (Trajectory Analysis) module of AMBER10 package.

Docking studies were done for all the 18 sialic acid analogues using Schrodinger (maestro). The protein is prepared by optimizing and minimizing the structure using Protein Preparation Wizard. The grid is generated using Receptor Grid Generation by picking the reference ligand which is already present in the PDB structure. HTVS is performed by importing 18 minimized sialic acid analogues using GLIDE module of Schrodinger software[8,9]. Induced fit is carried out for the five top scoring sialic acid analogues using Schrodinger to predict the ligand-induced conformational changes in receptor active sites[10].

## RESULTS

### Relative energy of neuraminic acid derivatives:

The relative energy is calculated for all the 10 neuraminic acid derivatives with respect to the absolute minimum energy of neuraminic acid (-2395.1 kcal/mol). The minimum energy conformations of neuraminic acid derivatives with respect to their relative energy are displayed in Table 3. Analogue 10 (5-N-acetyl-9-amino-9-deoxy neuraminic acid) is found to have the minimum energy equal to the minimum energy of neuraminic acid. Analogue 6 (2α-O-methyl-4-O-capriloyl-5-N-acetyl neuraminic acid) has the relative minimum energy of 14.7 kcal/mol.

It is noted from Table 3 that, in the minimum energy conformation for Analogue 10 (5-N-acetyl-9-amino-9-deoxy neuraminic acid), the side chain conformations (χ_1,_ χ_2_) prefer values around (-177.11°,-90.33°). For Analogue 6 (2α-O-methyl-4-O-capriloyl-5-N-acetyl neuraminic acid), the substituent dwelling side chain torsion angles (θ_1,_ θ_2_) and (β_1,_ β_2_) prefer the values (-69.48°,167.13°) and (-170.66°,-71.12°), respectively.

**TABLE 3:** MINIMUM ENERGY CONFORMATIONS OF NEURAMINIC ACID ANALOGUES WITH MULTIPLE MODIFICATIONS IN AQUEOUS ENVIRONMENT

Neuraminic acid derivative No	Relative energy kcal/mol	C1 substitution (γ_1_, γ_2_) (deg.)	C2 substitution (θ_1_,θ_2_) (deg.)	C4 substitution (β_1_, β_2_) (deg.)	C8 substitution (χ_1_, χ_3_) (deg.)	C9 substitution (χ_1_, χ_2_) (deg.)
N-acetyl Neuraminic Acid	0	-	-	-	-	-
1	49.1	(-52.53,-179.14)	-	-	-	-
2	73.4	(-70.20,171.27)	(-74.43,171.39)	-	-	-
3	35.6	(-100.06,179.65)	(-68.74,-178.68)	-	(175.31,49.82)	(175.31,-73.05)
4	76.7	(-73.32,177.19)	(-72.78,172.96)	(-169.32,-69.33)	(164.29,58.31)	(164.29,-65.13)
5	28.1	(-76.46,174.20)	(-63.98,174.14)	(-175.96,-73.03)	-	-
6	14.7	-	(-69.48,167.13)	(-170.66,-71.12)	-	-
7	115.9	(-68.53,178.43)	(-62.98,172.91)	(-177.16,-72.77)	(-175.04,47.84)	(-175.04,-75.38)
8	60.8	(-93.66,-174.97)	(-63.28,172.42)	(-174.64,-72.59)	-	-
9	75.4	-	(-72.59,175.08)	(-168.97,-74.63)	-	-
10	0	-	-	-	-	(-177.11,-90.33)

### Contribution of hydrogen bonds in structural stability:

**Fig. 1:**
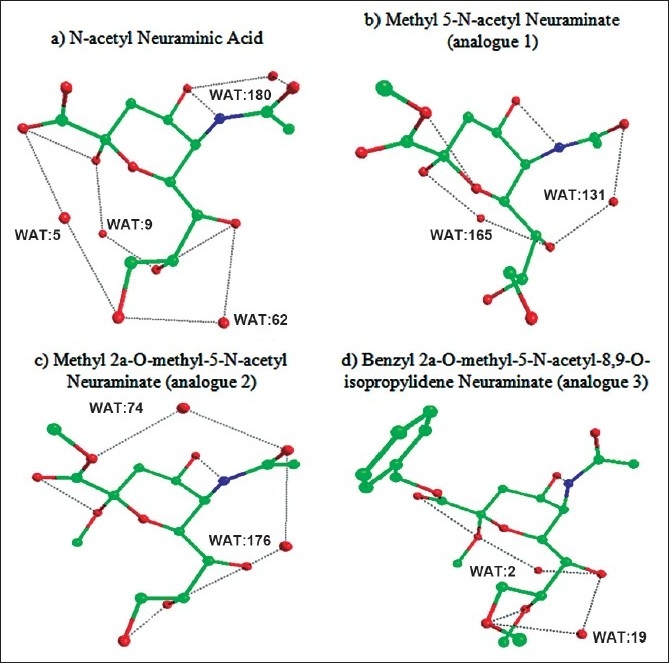
The 3D structure of neuraminic acid analogues in global minimum energy conformation state. (a) N-acetyl neuraminic acid. (b) methyl 5-N-acetyl neuraminate (c) methyl 2 α-O-methyl-5-N-acetyl neuraminate (d) benzyl 2α-Omethyl- 5-N-acetyl-8,9-O-isopropylidene.

**Fig. 2:**
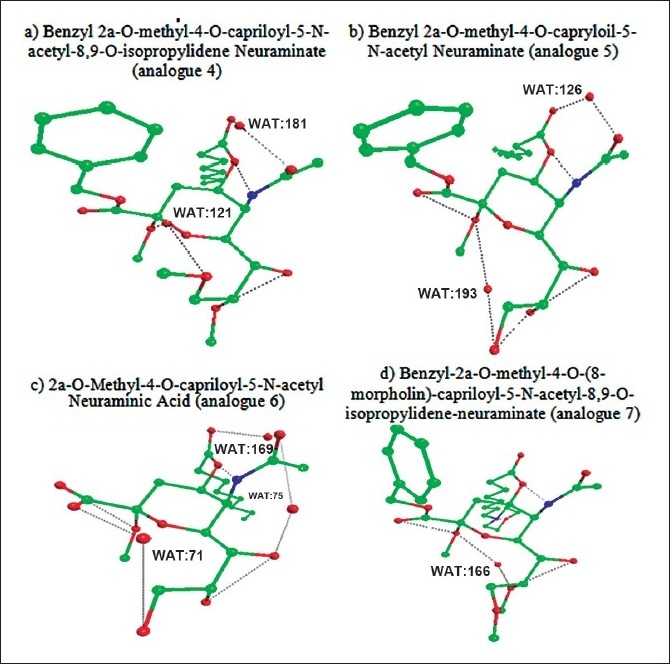
The 3D structure of neuraminic acid analogues in global minimum energy conformation state. (a) benzyl 2α-O-methyl-4-O-capriloyl-5-N-acetyl-8,9-Oisopropylidene neuraminate. (b) benzyl 2α-O-methyl-4-O-capriloyl- 5-N-acetyl-8,9-O-isopropylidene neuraminate (c) 2α-O-methyl-4-Ocapriloyl- 5-N-acetyl neuraminic acid (d) benzyl-2α-O-methyl-4-O-(8- morpholin)-capriloyl-5-N-acetyl-8,9-O-isopropylidene-neuraminate.

**Fig. 3:**
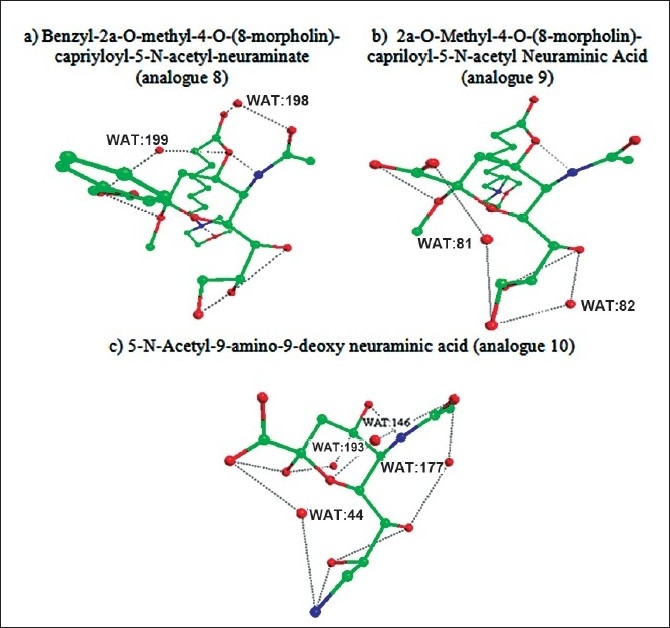
The 3D structure of neuraminic acid analogues in global minimum energy conformation state. (a) benzyl-2α-O-methyl-4-O-(8-morpholin)-capriyloyl-5-N-acetylneuraminate. (b) 2α-O-methyl-4-O-(8-morpholin)-capriloyl-5-Nacetyl neuraminic acid (c) N-acetyl-9-amino-9-deoxy neuraminic acid.

Figs. 1–3 showed the formation of water mediated hydrogen bonds and direct hydrogen bonds in neuraminic acid derivatives. It is well known from the previous findings that water mediated hydrogen bonds formed within the molecule plays a major role in stabilization of the molecule[11]. The water-mediated hydrogen bonds and direct hydrogen bonds are shown in Table 4. Comparison of water mediated hydrogen bonds with direct hydrogen bonds revealed that analogue 10 (5-N-acetyl-9-amino-9-deoxy neuraminic acid) has 4 water mediated hydrogen bonds and 4 direct hydrogen bonds. The nitrogen atom from the substituted NH_2_ group contributed to both water mediated and direct hydrogen bond. It is also observed that analogue 6 (2α-O-methyl-4-O-capriloyl-5-N-acetyl neuraminic acid) has three water mediated hydrogen bonds and three direct hydrogen bonds. It is inferred that the maximum number of direct and water mediated hydrogen bonds present in each neuraminic acid derivatives are responsible for the minimum energy and its stabilization of the molecule.

**TABLE 4:** HYDROGEN BONDS IN EACH NEURAMINIC ACID ANALOGUE WITH MULTIPLE MODIFICATIONS

Sialic acid derivative	Interacting sialic acid derivative atom 1	Mediating water	Distance (Å)	Interacting sialic acid derivative atom2	Distance (Å)
N-acetyl neuraminic Acid	O10	WAT:180	2.77	O4	2.97
	O2	WAT:9	2.91	O8	3.01
	O1B	WAT:5	3.19	O9	3.01
	O9	WAT:62	3.10	O7	3.08
	O8			O7	3.01
	O2			O1B	2.85
	O4			N5	2.93
methyl 5-N-acetyl neuraminate	O10	WAT:131	2.88	O7	2.74
	O2	WAT: 165	3.17	O7	3.10
	O4			N5	2.81
	O2			O1B	2.79
methyl 2α-0-methyl-5-N-acetyl neuraminate	O7	WAT: 176	2.60	O10	3.07
	O1A	WAT: 74	2.85	O10	3.03
	O2			O1B	2.82
	O8			O9	3.02
	O8			O7	2.96
	O4			N5	2.99
benzyl 2α-0-methyl-5-N-acetyl-8,9-0-isopropylidene neuraminate	O7	WAT: 19	2.67	O9	3.13
	O7	WAT: 2	3.14	O2	2.81
	O2			O1B	2.94
	O4			N5	3.01
	O8			O9	3.21
benzyl 2α-0-methyl-4-0-capriloyl-5-II-acetyl-8,9-0-isopropylidene neuraminate	O10	WAT: 181	2.90	O11*	3.17
	O9	WAT: 121	2.86	O2	2.88
	N5			O4	2.92
	O8			O7	2.81
benzyl 2α-0-methyl-4-0-capryloil-5-ll-acetyl neuraminate	O2	WAT: 193	3.03	O9	2.71
	O10	WAT: 126	2.48	O11	3.19
	O7			O8	2.91
	O8			O9	2.90
	O2			O1B	2.80
	N5			O4	2.87
2α-0-methyl-4-0-capriloyl-5-N-acetyl neuraminic acid	O7	WAT: 75	2.59	O10	2.81
	O10	WAT: 169	2.87	O11*	2.80
	O1A	WAT: 71	3.20	O9	3.07
	N5			O4	2.82
	O2			O1B	3.06
	O7			O8	2.95
benzyl-2α-0-methyl-4-0-(8-morpholin)-capriloyl-5-N-acetyl-8,9-O-isopropylidene-neuraminate	O8	WAT: 166	2.65	O2	3.08
	N18*			O20*	2.87
	N5			O4	2.86
	O2			O1B	2.84
	O7			O8	2.98
benzyl-2α-0-methyl-4-0-(8-morpholin)-capriytoyl-5-N-acetyl-neuraminate	O1B	WAT: 199	3.07	O4	2.93
	O11*	WAT: 198	3.03	O10	2.42
	O8			O7	2.88
	O9			O8	3.13
	O2			O1B	2.90
	N5			O4	2.78
	N18*			O20*	2.88
2α-0-methyl-4-0-(8-morpholin)-capriloyl-5-ll-acetyl neuraminic acid	O9	WAT: 82	3.04	O7	2.48
	O9	WAT:81	2.89	O1A	2.83
	N18*			O20*	2.88
	O8			O7	2.96
	O2			O1B	2.87
	O4			N5	2.85
5-H-acetyl-9-amino-9-deoxy neuraminic acid	O1B	WAT:44	2.75	N9*	3.10
	O10	WAT: 177	3.14	O7	2.90
	O4	WAT: 193	3.04	O2	2.68
	O5	WAT: 146	2.71	O10	2.89
	O8			N9*	2.52
	O7			O8	3.21
	N5			O4	3.05
	O2			O1B	2.81

* is the atom from the substituent group.

### Molecular dynamics of sialic acid analogues:

To study the conformational dynamics of the neuraminic acid derivatives, a 2ns molecular dynamics simulation was carried out. An in-depth analysis on the conformational features of all the 10 neuraminic acid analogues was done by collecting the frames for every 1ps.

### C-1 substituted side chain conformation:

**Fig. 4:**
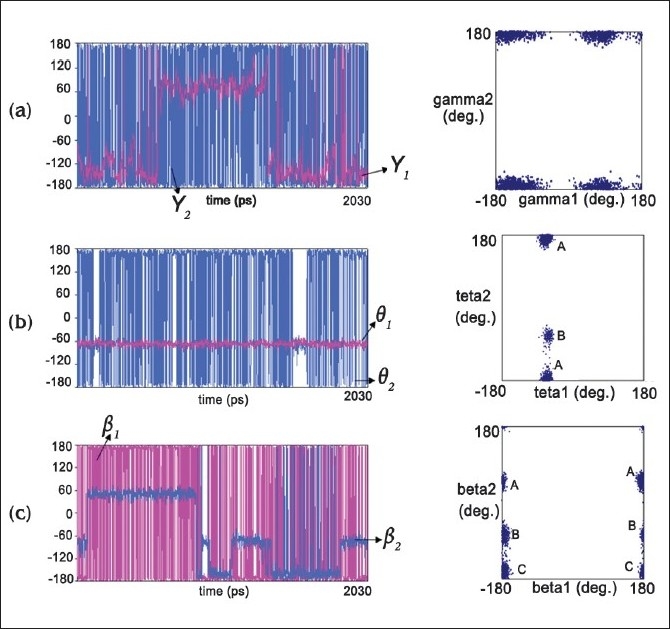
Molecular dynamics trajectory and the distribution plots of sialic acid analogues (a) γ_1_ V_s_ γ_2_ plots for benzyl 2α-O-methyl-4-O-capryloil-5-N-acetyl neuraminate, (b) θ_1_ Vs θ_2_ distribution plots for 2α-O-methyl-4-Ocapriloyl-5-N-acetyl neuraminic acid (c) β_1_V_s_ β_2_ plots for 2α-O-methyl-4-O-capriloyl-5-N-acetyl neuraminic acid.

For analogue 1 (methyl 5-N-acetyl neuraminate), the dihedral angles γ_1_ and γ_2_ exhibit a bifurcation throughout the molecular dynamics simulation. The torsional angle γ_1_ of analogue 2 (methyl 2α-O-methyl-5-N-acetyl neuraminate), analogue 3 (benzyl 2α-O-methyl-5-N-acetyl-8,9-O-isopropylidene neuraminate), analogue 4 (benzyl 2α-O-methyl-4-O-capriloyl-5-N-acetyl-8,9-O-isopropylidene neuraminate), analogue 5 (benzyl 2α-O-methyl-4-O-capryloil-5-N-acetyl neuraminate) and analogue 8 (benzyl-2α-O-methyl-4-O-(8-morpholin)-capriyloyl-5-N-acetyl-neuraminate) shows good distribution throughout the MD simulation, however γ_2_ is rigid in +180° and -180° regions. The shift of γ_1_ from -180° region to +70° region results in the energy decrease of 5 kcal/mol. Fig. 4a shows the molecular dynamic trajectory showing transitions of the substituent holding side chain torsion angles γ_1_ and γ_2_ along with (γ_1,_ γ_2_) distribution plot of analogue 5 (benzyl 2α-O-methyl-4-O-capryloil-5-N-acetyl neuraminate). In the case of analogue 7 (benzyl-2α-O-methyl-4-O-(8-morpholin)-capriloyl-5-N-acetyl-8,9-O-isopropylidene-neuraminate), γ_1_ bifurcates in negative regions such as -180°, -120° and -60°, whereas γ_2_ prefers +180° and -180° region.

### C-2 substituted side chain conformation:

For analogue 2 (methyl 2α-O-methyl-5-N-acetyl neuraminate), 3 (benzyl 2α-O-methyl-5-N-acetyl-8,9-O-isopropylidene neuraminate), 4 (benzyl 2α-O-methyl-4-O-capriloyl-5-N-acetyl-8,9-O-isopropylidene neuraminate), 5 (benzyl 2α-O-methyl-4-O-capryloil-5-N-acetyl neuraminate), 6 (2α-O-methyl-4-O-capriloyl-5-N-acetyl neuraminic acid) and 7 (benzyl-2α-O-methyl-4-O-(8-morpholin)-capriloyl-5-N-acetyl-8,9-O-isopropylidene-neuraminate), θ_1_ is rigid in -60° region and θ_2_ prefers +180° and -180° regions. Fig. 4b represents the dynamic behavior of torsions θ_1_ and θ_2_ along with (θ_1,_θ_2_) distribution plot of 2α-O-methyl-4-O-capriloyl-5-N-acetyl neuraminic acid (analogue 6) in aqueous environment.

However the dihedral angle θ_2_ of analogue 3 (benzyl 2α-O-methyl-5-N-acetyl-8,9-O-isopropylidene neuraminate), analogue 5 (benzyl 2α-O-methyl-4-O-capryloil-5-N-acetyl neuraminate) and analogue 6 (2α-O-methyl-4-O-capriloyl-5-N-acetyl neuraminic acid) exhibits an additional bifurcation in -60° region. In the case of analogue 8 (benzyl-2α-O-methyl-4-O-(8-morpholin)-capriyloyl-5-N-acetyl-neuraminate), θ_1_ prefers -70° region and θ_2_ prefers +180°, -180° and -70° regions.

### C-4 substituted side chain conformation:

β_1_ of analogue 4 (benzyl 2α-O-methyl-4-O-capriloyl-5-N-acetyl-8,9-O-isopropylidene neuraminate), 5 (benzyl 2α-O-methyl-4-O-capryloil-5-N-acetyl neuraminate), 7 (benzyl-2α-O-methyl-4-O-(8-morpholin)-capriloyl-5-N-acetyl-8,9-O-isopropylidene-neuraminate), and 8 (benzyl-2α-O-methyl-4-O-(8-morpholin)-capriyloyl-5-N-acetyl-neuraminate) prefers +180° and -180° regions. β_2_ of analogue 5 (benzyl 2α-O-methyl-4-O-capryloil-5-N-acetyl neuraminate), 7 (benzyl-2α-O-methyl-4-O-(8-morpholin)-capriloyl-5-N-acetyl-8,9-O-isopropylidene-neuraminate) and 8 (benzyl-2 α-O-methyl-4-O-(8-morpholin)-capriyloyl-5-N-acetyl-neuraminate) prefers -180° and -70° regions. β_2_ of analogue 4 (benzyl 2α-O-methyl-4-O-capriloyl-5-N-acetyl-8,9-O-isopropylidene neuraminate) prefers -180°, -120°, +180° and -60° regions.

For analogue 7 (benzyl-2α-O-methyl-4-O-(8-morpholin)-capriloyl-5-N-acetyl-8,9-O-isopropylidene-neuraminate), the shift of β 2 from -70° to -180° region results in the energy decrease of up to 6 kcal/mol. In the case of analogue 6 (2α-O-methyl-4-O-capriloyl-5-N-acetyl neuraminic acid), β_2_ shows good distribution in -180°, +180°, -70° and +60° regions. β_1_ prefers +180° and -180° regions. Fig. 4c represents the dynamic behavior of torsions β_1_ and β_2_ along with (β_1,_β_2_) distribution plot of 2α -O-methyl-4-O-capriloyl-5-N-acetyl neuraminic acid (analogue 6) in aqueous environment.

### C-8 substituted side chain conformation:

**Fig. 5:**
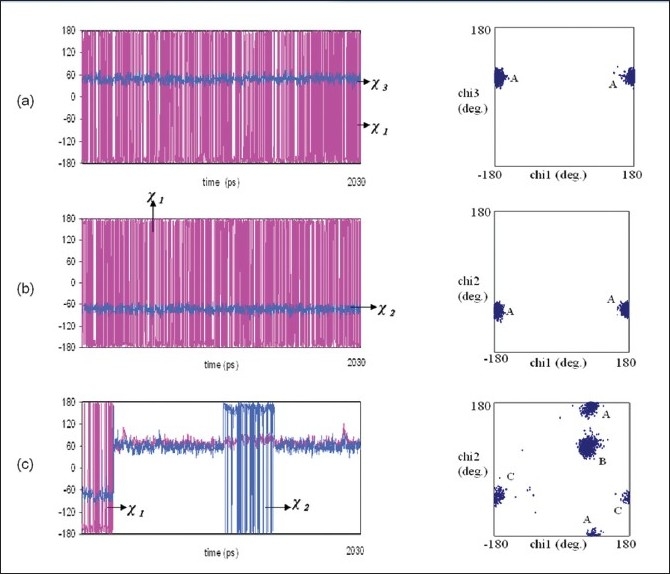
Molecular dynamics trajectory and the distribution plots (a) χ_1_ V_s_ χ_3_ plots for benzyl 2α-O-methyl-5-N-acetyl-8,9-Oisopropylidene neuraminate, (b) χ_1_ V_s_ χ_2_ plots for benzyl 2α-Omethyl-4-O-capriloyl-5-N-acetyl-8,9-O-isopropylidene neuraminate and (c) χ_1_ Vs χ_2_ plots for 5-N-Acetyl-9-amino-9-deoxy neuraminic acid

For analogue 3 (benzyl 2α-O-methyl-5-N-acetyl-8,9-O-isopropylidene neuraminate), analogue 4 (benzyl 2α-O-methyl-4-O-capriloyl-5-N-acetyl-8,9-O-isopropylidene neuraminate) and analogue 7 (benzyl-2α-O-methyl-4-O-(8-morpholin)-capriloyl-5-N-acetyl-8,9-O-isopropylidene-neuraminate), χ_1_ prefers +180° and -180° regions and χ_3_ is rigid in +70° region. Fig. 5a describes the molecular dynamic trajectory showing transitions of the different substituent holding side chain torsion angles χ_1_ and χ_3_ along with (χ_1,_χ_3_) distribution plot of analogue 3 (benzyl 2α-O-methyl-5-N-acetyl-8,9-O-isopropylidene neuraminate).

### C-9 substituted side chain conformation:

χ_1_ analogue 3 (benzyl 2α-O-methyl-5-N-acetyl-8,9-O-isopropylidene neuraminate), 4 (benzyl 2α-O-methyl-4-O-capriloyl-5-N-acetyl-8,9-O-isopropylidene neuraminate) and 7 (benzyl-2α-O-methyl-4-O-(8-morpholin)-capriloyl-5-N-acetyl-8,9-O-isopropylidene neuraminate) prefers +180° and -180° regions and χ_2_ prefers -70° region. In the case of analogue 10 (5-N-acetyl-9-amino-9-deoxy neuraminic acid), χ_1_ prefers -180° +60° and +180° regions and χ_2_ shows bifurcation in -70°, +70°, +180° and -180° regions. The shift of χ_1_ from -180° region to +60° region resulted in the energy decrease of up to 10kcal/mol. Fig. 5 (b and c) represents the dynamic behavior of torsions χ_1_ and χ_2_ along with (χ_1_, χ_2_) distribution plot of neuraminic acid analogues benzyl 2α-O-methyl-4-O-capriloyl-5-N-acetyl-8,9-O-isopropylidene neuraminate (analogue 4) and 5-N-acetyl-9-amino-9-deoxy neuraminic acid (analogue 10) in aqueous environment.

### Modeling of sialic acid analogues and Influenza hemagglutinin complexes:

**Fig. 6:**
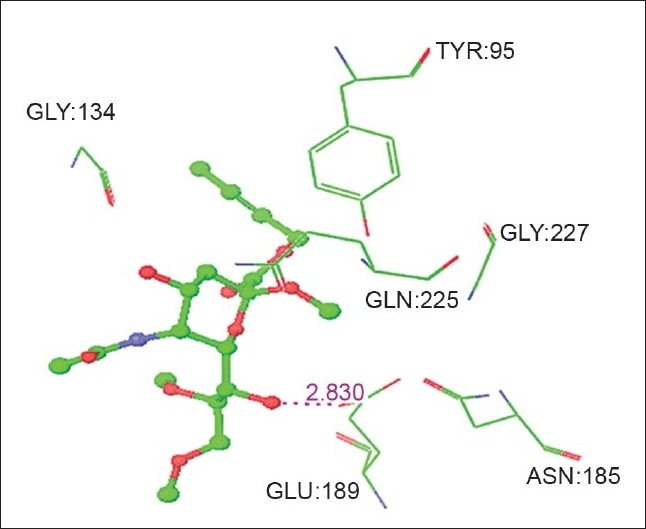
Sialic acid analogue (2α-O-methyl-5-N-acetyl-8,9-Oisopropylidene neuraminate) at the active site of Influenza Hemagglutinin

**Fig. 7:**
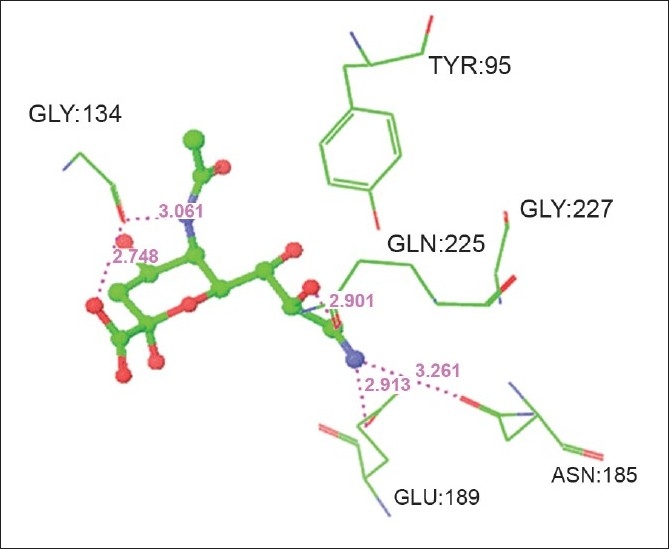
Sialic acid analogue (5-N-acetyl-9-amino-9-deoxy neuraminic acid) at the active site of Infl uenza Hemagglutinin

High Throughput Virtual Screening was done for ten neuraminic acid analogues to find out the structures (ligands) most likely to bind to the Influenza hemagglutinin. The top five ligands with best docking score and minimum energy are subjected to induced fit docking. The glide score along with the glide energy is displayed in Table 5. The inter-molecular interactions between the top five ligands and the Influenza hemagglutinin are noted and displayed in Table 6. Figs. 6 and 7 shows the neuraminic acid analogues 3 (benzyl 2α-O-methyl-5-N-acetyl-8,9-O-isopropylidene neuraminate) and 10 (5-N-acetyl-9-amino-9-deoxy neuraminic acid) at the active site of Influenza HA, respectively. The bound state conformations of the substituent holding side chains of sialic acid analogues are displayed in Table 7.

**TABLE 5:** GLIDE DOCKING SCORE AND GLIDE ENERGY OF INFLUENZA HEMAGGLUTININ - NEURAMINIC ACID COMPLEXES

Analogue no	Neuraminic acid derivative	Glide energy (kcal/mol)	Glide score
5	Benzyl 2a-O-methyl-4-O-capryloil-5-N-acetyl Neuraminate	-54.64	-8.64
10	5-N-Acetyl-9-amino-9-deoxy Neuraminic Acid	-36.70	-7.73
2	5-N-Acetyl-9-amino-9-deoxy Neuraminic Acid	-42.87	-7.71
4	Benzyl 2α-O-methyl-4-O-capriloyl-5-N-acetyl-8,9-O-isopropylidene Neuraminate	-45.91	-6.71
3	Benzyl 2a-O-methyl-5-N-acetyl-8,9-O-isopropylidene Neuraminate	-42.16	-5.34

**TABLE 6:** INTER-MOLECULAR INTERACTIONS BETWEEN THE NEURAMINIC ACID ANALOGUES AND INFLUENZA HEMAGGLUTININ

Analogue no	Neuraminic acid derivative	Ligand atom	Protein		Distance (Å)
			Residue	Atom	
5	Benzyl 2a-O-methyl-4-O-capryloil-5-N-acetyl Neuraminate	O8	GLU:225	OE1	2.677
		O9	GLU:225	OE1	2.698
		O9	GLY:227	N	2.338
10	5-N-Acetyl-9-amino-9-deoxy Neuraminic Acid	N	GLY:134	O	3.061
		O1	GLY:134	O	2.748
		N9	ASN:185	OD1	3.261
		N	GLU:189	OE1	2.913
		O10	GLN:225	OE1	2.901
2	Methyl 2α-O-methyl-5-N-acetyl Neuraminate	O9	GLY:134	O	2.798
		O2	GLU:189	OE1	2.677
		O1	TYR:95	OH	2.675
3	Benzyl 2a-O-methyl-5-N-acetyl-8,9-O-isopropylidene Neuraminate	O7	GLU:189	OE1	2.830
4	Benzyl 2α-O-methyl-4-O-capriloyl-5-N-acetyl-8,9-O-isopropylidene Neuraminate	O9	GLU:225	OE1	2.743
		O7	GLY:134	OE1	2.991
		O4	GLU:225	O	2.936

**TABLE 7:** BOUND STATE CONFORMATIONS OF THE SUBSTITUENT HOLDING SIDE CHAINS OF SIALIC ACID ANALOGUES

Neuraminic acid Analogue No	C1 substitution (γ_1_, γ_2_) (deg.)	C2 substitution (θ_1_,θ_2_) (deg.)	C4 substitution (β _1_, β _2_) (deg.)	C8 substitution (χ _1_, χ _3_) (deg.)	C9 substitution (χ _1_, χ _2_) (deg.)
	-	-	-	-	-
2	(47.8,-179.9)	(-66.4,-178.7)	-	-	-
3	(92.0,171.9)	(-74.7,166.5)	-	(102.9,-63.0)	(102.9,-176.5)
4	(48.1,179.7)	(-63.7,-54.1)	(-178.0,-74.1)	(-171.4,50.3)	(-171.4,-75.0)
5	(13.8,158.0)	(-66.1,-138.7)	(-172.8,-63.6)	-	-
10	-	-	-	-	(-173.5,96.1)

## DISCUSSION

The current study reveals the probable conformational models for neuraminic acid derivatives with multiple substitutions at positions C-1/C-2/C-4/C-8/C-9 in aqueous environment. Water mediated hydrogen bonding interaction plays a dominant role in stabilizing the conformational structures of these neuraminic acid derivatives. The accessible conformations for neuraminic acid analogues with multiple substituents holding side chain linkages observed by the present MD study correlate well with those reported for similar linkages in various Neu5Ac-α2→8-Neu5Ac moiety present in all the di- and tri-sialogangliosides by earlier studies[11,12]. Present MD results show a dynamic behaviour for χ_1_ of analogue 10 (5-N-acetyl-9-amino-9-deoxy neuraminic acid) at the cost of 10 kcal/mol (Fig. 5c), which is a vivid indication that this molecule prefers -60° region. Analogue 10 is observed to have the lowest energy of -2395.1 kcal/mol when compared to other neuraminic acid analogues with multiple substituents and it shows good results in hydrogen bonding interactions with four water mediated hydrogen bonds and four direct hydrogen bonds. The nitrogen from the substituted NH_2_ group contributes to both water mediated and direct hydrogen bond. Docking studies show the mode of binding of sialic acid derivatives into the binding pocket of Influenza hemagglutinin which in turn explains their order of specificity. It is very interesting to know that the neuraminic acid analogues, benzyl 2α -O-methyl-4-O-capryloil-5-N-acetyl neuraminate (analogue 5), 5-N-acetyl-9-amino-9-deoxy neuraminic acid (analogue 10), 5-N-acetyl-9-amino-9-deoxy neuraminic acid (analogue 2), benzyl 2α-O-methyl-4-O-capriloyl-5-N-acetyl-8,9-O-isopropylidene neuraminate (analogue 4) and benzyl 2α-O-methyl-5-N-acetyl-8,9-O-isopropylidene neuraminate (analogue 3) have greater docking score than the co-crystal ligand (PDB reference ligand) such as -8.64, -7.73, -7.71, -6.71 and -5.34, respectively and minimum energy such as -54.64 kcal/mol, -36.70 kcal/mol, -42.87 kcal/mol, -45.91 kcal/mol and -42.16 kcal/mol, respectively.

The present study provides accessible conformational models for synthetic neuraminic acid analogues with multiple substituents at positions C-1, C-2, C-4, C-8 or C-1 in aqueous environment. Direct and water mediated hydrogen bonding schemes greatly involve in stabilizing the three dimensional conformational structures of these neuraminic acid analogues. This study also shows the dynamics trajectory and distribution plot for the substituent holding side chain linkages of the neuraminic acid analogues. The high affinity inhibitors modeled in this study saturate the hemagglutinin (HA) receptor[5] and can be used as potential antiinfluenza drugs.
